# Molecular and Biochemical Mechanism of Cannabidiol in the Management of the Inflammatory and Oxidative Processes Associated with Endometriosis

**DOI:** 10.3390/ijms23105427

**Published:** 2022-05-12

**Authors:** Tiziana Genovese, Marika Cordaro, Rosalba Siracusa, Daniela Impellizzeri, Sebastiano Caudullo, Emanuela Raffone, Francesco Macrí, Livia Interdonato, Enrico Gugliandolo, Claudia Interlandi, Rosalia Crupi, Ramona D’Amico, Roberta Fusco, Salvatore Cuzzocrea, Rosanna Di Paola

**Affiliations:** 1 Department of Chemical, Biological, Pharmaceutical and Environmental Sciences, University of Messina, Viale Ferdinando Stagno D’Alcontres 31, 98166 Messina, Italy; tgenovese@unime.it (T.G.); rsiracusa@unime.it (R.S.); dimpellizzeri@unime.it (D.I.); linterdonato@unime.it (L.I.); salvator@unime.it (S.C.); 2 Department of Biomedical, Dental and Morphological and Functional Imaging, University of Messina, Via Consolare Valeria, 98125 Messina, Italy; marika.cordaro@unime.it; 3 Papardo Hospital, 98166 Messina, Italy; sebastianocaudullo@aopapardo.it; 4 Multi-Specialist Istitute Rizzo, Torregrotta, 98043 Messina, Italy; emanuelaraffone@virgilio.it; 5 Department of Veterinary Sciences, University of Messina, 98168 Messina, Italy; fmacri@unime.it (F.M.); egugliandolo@unime.it (E.G.); cinterlandi@unime.it (C.I.); rcrupi@unime.it (R.C.); dipaolar@unime.it (R.D.P.); 6 Department of Clinical and Experimental Medicine, University of Messina, Via Consolare Valeria, 98125 Messina, Italy

**Keywords:** endometriosis, cannabidiol, inflammation

## Abstract

Endometriosis is usually associated with inflammation and chronic pelvic pain. This paper focuses the attention on the anti-inflammatory, anti-oxidant and analgesic effects of cannabidiol (CBD) and on its potential role in endometriosis. We employed an in vivo model of endometriosis and administered CBD daily by gavage. CBD administration strongly reduced lesions diameter, volume and area. In particular, it was able to modify lesion morphology, reducing epithelial glands and stroma. CBD showed anti-oxidant effects reducing lipid peroxidation, the expression of Nox-1 and Nox-4 enzymes. CBD restored the oxidative equilibrium of the endogenous cellular defense as showed by the SOD activity and the GSH levels in the lesions. CBD also showed important antifibrotic effects as showed by the Masson trichrome staining and by downregulated expression of MMP-9, iNOS and TGF-β. CBD was able to reduce inflammation both in the harvested lesions, as showed by the increased Ikb-α and reduced COX2 cytosolic expressions and reduced NFkB nuclear localization, and in the peritoneal fluids as showed by the decreased TNF-α, PGE2 and IL-1α levels. CBD has important analgesic effects as showed by the reduced mast cells recruitment in the spinal cord and the reduced release of neuro-sensitizing and pro-inflammatory mediators. In conclusion, the collected data showed that CBD has an effective and coordinated effects in endometriosis suppression.

## 1. Introduction

Endometriosis, a chronic condition in which the endometrium, which usually provides the inner facing of the uterus, sprouts in other areas, normally on the bowel, ovaries, bladder, rectum, and pelvic lining. Depending on the stage of the disease, it could lead to dysmenorrhoea, infertility and chronic recurring pelvic pain in billions of women of reproductive age [1]. Retrograde menstruation is the generally accepted mechanism underlying the pathogenesis of endometriosis [2]. This mechanism was originally proposed in 1927, whereby endometrial fragments migrate from the fallopian tubes into the peritoneal cavity during menstruation. Once the endometrial debris becomes ectopic, adhesion needs to occur in order to initiate the development of lesions and the induction of endometriosis. While the mechanisms underlying this process remain unclear, it is considered that immune dysfunction and the subsequent inability to effectively clear these fragments enables endometrial lesions to form in the peritoneal cavity [3]. From the histological point of view, epithelial cells and stroma are capsuled in surrounding tissue and show extensive fibrosis and smooth muscle metaplasia. Lesions are characterized by invasiveness and mobility, fibroblast–myofibroblast differentiation and epithelial–mesenchymal transition. Recent findings underline the importance of the oxidative imbalance and inflammatory responses both at the lesion site and in the peritoneum and the related chronic pain state. These proinflammatory microenvironment includes inflammatory cytokines/chemokines, prostaglandins, growth factors (GR) and reactive oxygen species (ROS). In peritoneal fluid of patients, increased levels of protein oxidative stress markers, tumor necrosis factor-α (TNF-α), prostaglandin E2 (PGE_2_) and interleukin (IL) IL-1β and IL-8 were found [4,5,6,7]. These mediators produce activation of sensory nerve and nociceptive pathways [4,8,9], proposing inflammatory mechanisms may be critical in endometriosis associated pain [10,11]. Further retrograde menstruations increase extra-uterine debris and lesions. The increased inflammatory answer within the peritoneum activates sensory nerves to induce chronic pelvic pain [12]. Moreover, stimulation of sensory afferent nerves leads to the recruitment of mast cells and consequent release of the previously mentioned proinflammatory mediators, which contributes to establishing a positive feedback loop called “neurogenic inflammation” [13]. The activation of peripheral nerve endings translates the stimuli to the spinal cord inducing central sensitization. Cannabinoid receptor and cannabinoids are potential targets for pain and inflammation [14,15,16]. Δ9-tetrahydrocannabinol (THC) and cannabidiol (CBD) are the primary compounds contained in *Cannabis sativa*, although it contains more approximately 80 different cannabinoids [17]. CBD, structurally related to THC, is a non-psychoactive compound with therapeutic potential for inflammation, cancer and neuropathic pain [18,19,20]. Recent papers discussed the effects of CBD consumption in endometriosis focusing the attention on the pelvic pain and related symptoms [21,22]. CBD appears to be effective across all reported symptoms, with a noted propensity for inhaled delivery due to the potential increased speed of onset of effects versus the slower onset of oral products, particularly for pelvic pain. Conversely, oral forms appeared to be superior for the less reported mood and gastrointestinal categories. Whilst topical products demonstrated a good effect on pain, due to a very small data set, caution should be exercised in interpreting or extrapolating from this data.

Basic and/or clinical studies have shown that cannabidiol has multidirectional properties, such as antioxidant, anti-inflammatory [23], immunomodulatory, antiarthritic, anticonvulsant, neuroprotective [24], precognitive [25], anti-anxiety, antipsychotic and anti-proliferative, among others [25]. Thus, CBD possesses wide therapeutic potential, which includes e.g., hypertension [26,27], epilepsy, neurodegenerative diseases (multiple sclerosis, Alzheimer’s, Parkinson’s and Huntington’s diseases) [25,28], neuropsychiatric disorders (depression, anxiety disorders, schizophrenia, post-traumatic stress disorder, autistic spectrum disorders) [25,29], gastrointestinal disorders (nausea and vomiting, inflammatory bowel diseases, irritable bowel syndrome) [30], rheumatic diseases [26], graft versus host disease and cancer [28,31]. However, most of these indications require further investigation to confirm clinical effectiveness.

This paper was aimed to evaluate the molecular properties of CBD in an in vivo model of endometriosis underlying its regulatory impact on the pathways involved in the pathology.

## 2. Results

### 2.1. Effect of CBD Administration on Endometriotic Lesions

**Figure 1:**
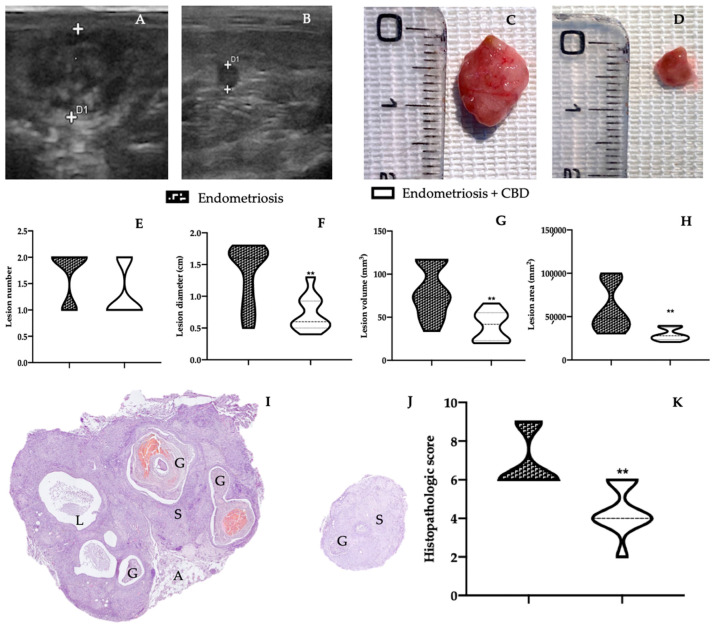
CBD administration reduced lesion size endometriosis-induced: hfUS analysis: vehicle (**A**), CBD (**B**), macroscopic analysis: vehicle (**C**), CBD (**D**), lesion number (**E**), lesion diameter (**F**), lesion volume (**G**), lesion area (**H**), histological analysis: vehicle (**I**), CBD (**J**), histopathological score (**K**): G: glands; S: stroma; L: cyst lumen; A: adipose tissue. For each analysis, *n* = 5 animals were employed. For the histological analysis, *n* = 5 different fields from *n* = 5 different animals were evaluated. ** *p* < 0.01 vs. endometriosis.

At the end of the experiment, pelvic ultrasound was employed to evaluate the presence of the endometriomas. The evaluation included both anterior and posterior pelvic compartments to evaluate the different endometriosis locations. Lesions were detected in the inner surface of the peritoneal cavity in both vehicle (Figure 1A) and CBD (Figure 1B) groups. The lesions of both groups appeared flat or nodular, like a plaque. Lesions from the CBD group appeared smaller and more superficially attached to the peritoneal cavity. The macroscopic analysis (Figure 1C,D) confirmed the hfUS examination. The two groups did not differ for the lesions number (Figure 1E), but diameter (Figure 1F), volume (Figure 1G) and area (Figure 1H) were significantly smaller in lesions harvested from the vehicle group (Figure 1C), as compared to the one harvested from the CBD group (Figure 1D). The histological analysis displayed that CBD administration also changed lesions morphology (Figure 1K). Lesions from vehicle treated rats showed characteristic endometrial glands and stroma (Figure 1I), which were significantly reduced in the histopathological marks of endometriosis (Figure 1J,K).

### 2.2. Effect of CBD Administration on Oxidative Stress Associated with Endometriosis

**Figure 2:**
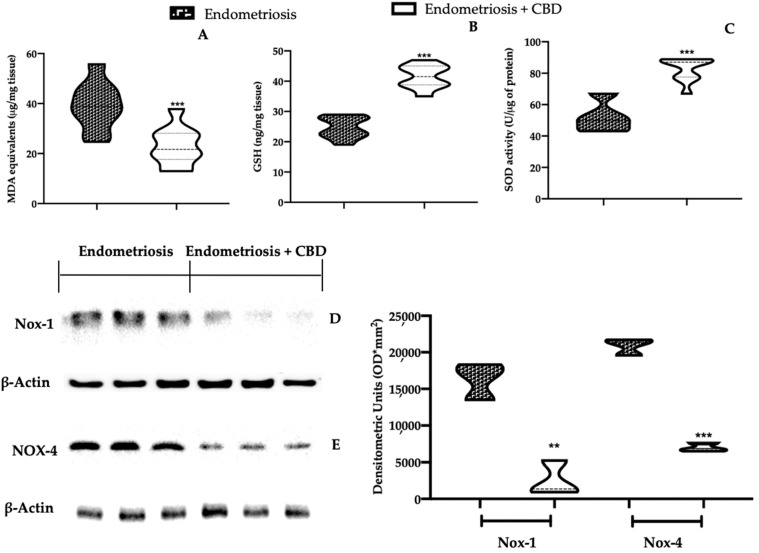
CBD administration reduced pro-oxidative alteration in endometriotic lesions: malondialdehyde (MDA) levels (**A**), GSH levels (**B**), SOD activity (**C**), Western blot analysis of Nox-1 (**D**) and Nox-4 (**E**) expression. For each analysis, *n* = 5 animals were employed. ** *p* < 0.01 vs. endometriosis, *** *p* < 0.001 vs. endometriosis.

The anti-oxidant effects of CBD administration were evaluated by biochemical and Western blot analysis. The thiobarbituric acid reactive substances (TBARS) test showed an increased lipid peroxidation in lesions harvested from vehicle treated rats, while tissues harvested from CBD rats showed a reduced membrane peroxidation (Figure 2A). Moreover, CBD administration restored the endogenous cellular defense mechanisms increasing glutathione (GSH) levels (Figure 2B) and superoxide dismutase (SOD) activity (Figure 2C). Western blot analysis showed increased nicotinamide adenine dinucleotide phosphate (NADPH) oxidases (Nox) 1 (Figure 2D) and Nox-4 (Figure 2E) expressions in lesions from vehicle treated rats while CBD administration significantly reduced their expressions. 

### 2.3. Effect of CBD Administration on Fibrosis Associated with Endometriosis

**Figure 3:**
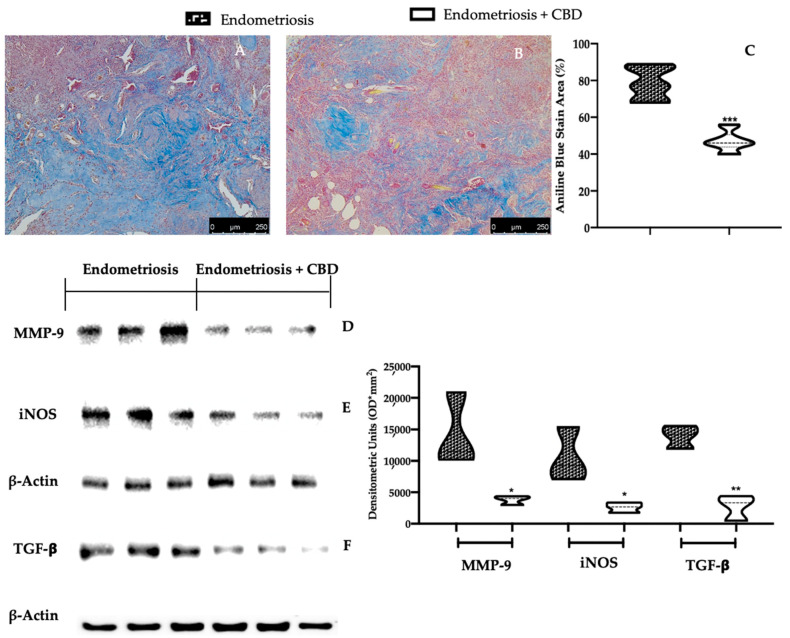
CBD administration reduced fibrosis endometriosis-induced: Masson trichrome staining: vehicle (**A**), CBD (**B**), aniline blue stain area (**C**); Western blot analysis of MMP-9 (**D**), iNOS (**E**) and TGF-β expression (**F**). For each analysis, *n* = 5 animals were employed. For the Masson trichrome staining, *n* = 5 different fields from *n* = 5 different animals were evaluated. * *p* < 0.05 vs. endometriosis, ** *p* < 0.01 vs. endometriosis, *** *p* < 0.001 vs. endometriosis.

CBD administration also showed important anti-fibrotic effects. Masson trichrome staining showed a reduction of collagen fibers in lesions from CBD treated rats (Figure 3B,C), as compared to the vehicle one (Figure 3A,C). Well in line with the staining, Western blot analysis confirmed this anti-fibrotic effect as showed by the reduced expression of matrix metallopeptidase 9 (MMP-9) (Figure 3D), inducible nitric oxide synthase (iNOS) (Figure 3E) and transforming growth factor-β (TGF-β) (Figure 3F) in CBD tissues, as compared to the vehicle treated rats. 

### 2.4. Effect of CBD Administration on Inflammation with Endometriosis

**Figure 4:**
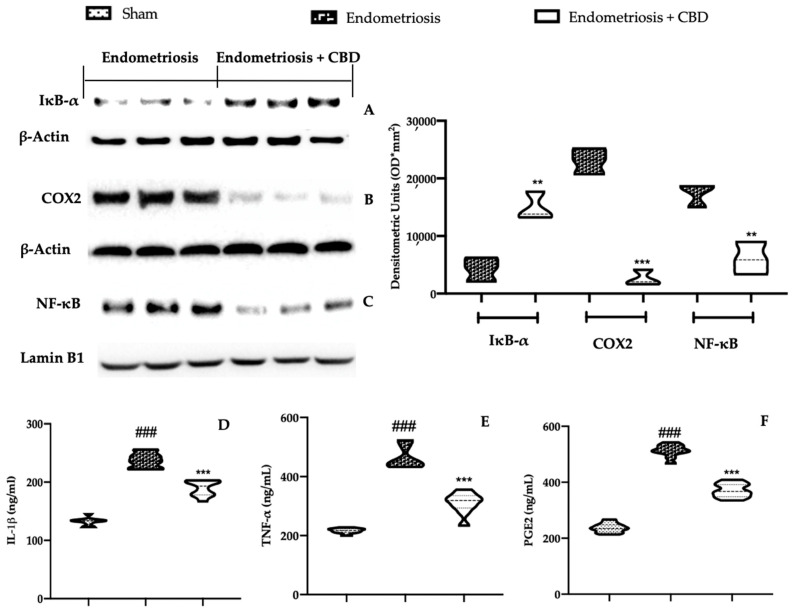
CBD administration reduced the pro-inflammatory state endometriosis-induced: Western blot analysis of Ikb-α (**A**), COX2 (**B**), NF-kB (**C**), IL-1β (**D**), TNF-α (**E**) and PGE_2_ (**F**) levels in the peritoneal fluids. For each analysis, *n* = 5 animals were employed. ** *p* < 0.01 vs. endometriosis, *** *p* < 0.001 vs. endometriosis, ### *p* < 0.001 vs. sham.

The anti-inflammatory effects of CBD were assessed by Western blot analysis. Increased nuclear factor of kappa light polypeptide gene enhancer in B-cells inhibitor alpha (Ikb-α) (Figure 4A) and reduced cyclooxygenase-2 (COX2) (Figure 4B) cytosolic expressions were found in samples harvested from CBD rats, as compared with the vehicle one. Additionally, a nuclear factor kappa light chain enhancer of activated B cells (NF-kB) expression was found to be reduced in the harvested lesions from CBD administered rats, as compared to the vehicle rats (Figure 4C). Endometriosis induced an increased inflammatory state in peritoneal fluid, as compared to the sham group. IL-1β (Figure 4D), TNF-α (Figure 4E) and PGE_2_ (Figure 4F) levels were found to be reduced in the peritoneal fluids of rats treated with CBD. 

### 2.5. Effect of CBD Administration on Mast Cells Recruitment and Pain-Related Mediators Associated with Endometriosis

**Figure 5:**
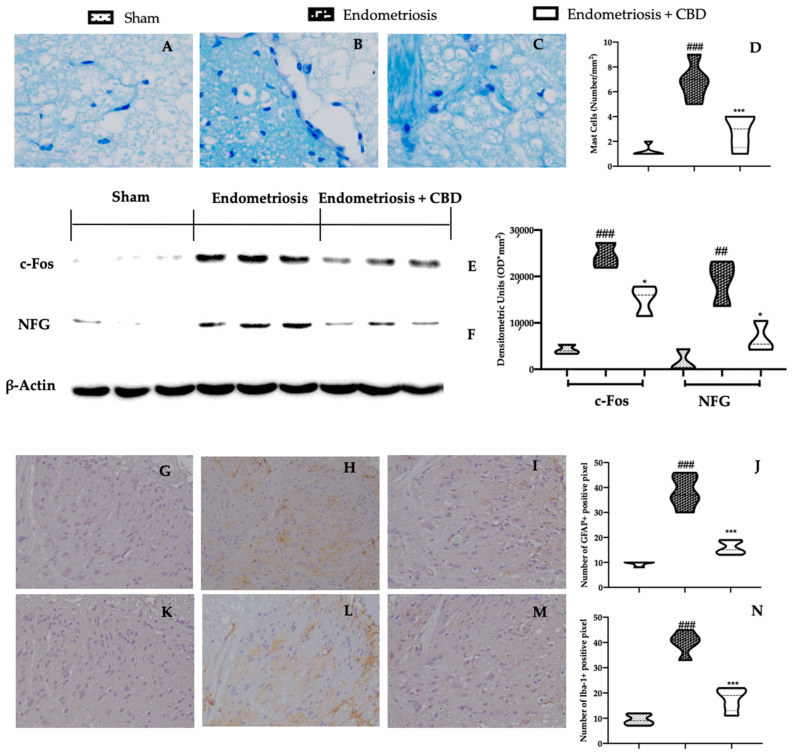
CBD administration reduced pain-mediators and neuroinflammatory state endometriosis-induced: Toluidine blue staining: sham (**A**), vehicle (**B**), CBD (**C**), mast cell number (**D**), Western blot analysis of c-FOS (**E**), and NGF (**F**) expression; Immunohistochemical analysis of GFAP: sham (**G**), vehicle (**H**), CBD (**I**), graphical quantification of GFAP expression (**J**), immunohistochemical analysis of Iba-1: sham (**K**), vehicle (**L**), CBD (**M**), graphical quantification of Iba-1 expression (**N**). For each analysis, *n* = 5 animals were employed. For the toluidine blue staining and the immunohistochemical analysis, *n* = 5 different fields from *n* = 5 different animals were evaluated. * *p* < 0.05 vs. endometriosis, ## *p* < 0.01 vs. sham, *** *p* < 0.001 vs. endometriosis, ### *p* < 0.001 vs. sham. Scale bar 100 μm.

Furthermore, it has been shown that a significant increase in mast cell number in the spinal cord of rats subjected to endometriosis and treated with vehicle (Figure 5B,D), as compared to sham animals (Figure 5A,D). CBD administration strongly reduced mast cells recruitment in the spinal cord (Figure 5C,D). The reduced mast cells infiltration also relates with the decreased expression of the neurogenic mediators c-FOS (Figure 5E) and nerve growth factor (NGF) (Figure 5F) in the spinal cord of rats treated with CBD, as compared to the vehicle animals. Additionally, immunohistochemical analysis showed increased glial fibrillary acidic protein (GFAP) and ionized calcium binding adaptor molecule 1 (Iba-1) expression in the spinal cord harvested from vehicle treated rats (Figure 5H,J,L respectively), as compared to the sham animals (Figure 5G,J,K,L respectively). Tissues harvested from CBD-treated rats showed reduced expression of both neuroinflammatory markers (Figure 5I,J,M,N).

### 2.6. Effect of CBD Administration on Pain Sensitivity Threshold Associated with Endometriosis

**Figure 6:**
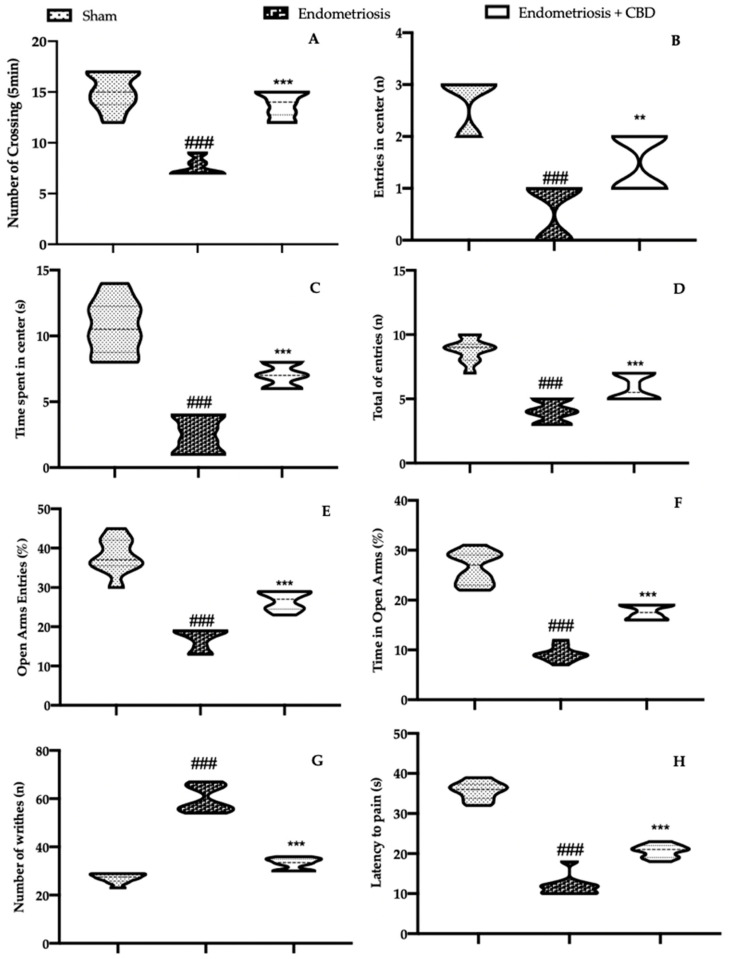
CBD administration reduced pain-behavior endometriosis-induced: open field test: number of crossings (**A**), number of entries in central square (**B**), and time spent in central square (**C**), elevated plus maze test: number of entries in closed and open arms (**D**), % of open entries (**E**), % of time in open arms (**F**), acetic-acid-induced abdominal contractions (**G**), hot plate test (**H**). For each analysis, *n* = 5 animals were employed. ** *p* < 0.01 vs. endometriosis, *** *p* < 0.001 vs. sham, ### *p* < 0.001 vs. endometriosis.

Endometriosis is associated with behavioral alterations-pain induced. CBD administration ameliorated exploratory behavior and locomotor activity, as compared to the vehicle treated rats (Figure 6A–C). Additionally, rats treated with CBD and tested in the elevated plus maze test showed a reduced number of entries in closed and open arms (Figure 6D), % of open entries (Figure 6E) and the % of time in open (Figure 6F), as compared to the vehicle treated animals. Vehicle treated animals showed increased sensitivity to the acetic-acid-induced abdominal contractions (Figure 6G) and to thermal stimuli (Figure 6H), which were significantly reduced by CBD administration (Figure 6G,H).

## 3. Discussion

Molecular targets, pharmacokinetics, and safety and abuse liability of CBD have been widely discussed [32]. It is the primary non-addictive and non-euphorizing compound of cannabis and displayed therapeutic potential for healing a wide range of disorders including chronic inflammation [33], chronic pain [22,29], epilepsy [34,35], CNS disorders [36,37,38] and neuropsychiatric disorders [39]. Additionally, previous authors evaluated the effects of CBD in endometriosis [22,40,41,42]. 

This paper showed that the anti-oxidant, antifibrotic and anti-inflammatory activities of CBD would be useful to counteract the development of endometriosis and related chronic pain. CBD administration strongly reduced cyst diameter, volume and area. In particular, it was able to modify lesion morphology, reducing epithelial glands and stroma. 

From the molecular point of view, endometriosis is characterized by nitrosative and oxidative stress [43]. ROS produces free radicals and oxidizing agent that induce a cascade of reactions that produce pro-inflammatory mediators and growth factors that may stimulate the pathogenesis of fibrosis [44]. These ROS are released by the electron transport chain reactions and enzymes like COXs, oxidoreductases, peroxisomal oxidases, lipoxygenases and NADPH oxidases are attributed to ROS synthesis [45]. CBD reduced lipid peroxidation and restored the oxidative equilibrium in the endogenous cellular defense as showed by the SOD activity and the GSH levels in the lesions. Additionally, CBD reduced Nox-1 and Nox-4 expressions, enzymes that play a key role in the synthesis of H_2_O_2_ and O_2_^.^ [46]. Nox-4 overexpression has also been related with TGF-β expression and fibrosis [47]. Masson trichrome staining showed a significant reduction of collagen deposition in animals treated with CBD. The reduced fibrosis was also confirmed by the downregulation of the MMP-9, iNOS and TGF-β expression, which were significantly increased in endometriotic lesions. The increased oxidative stress and fibrosis are accompanied with an increased inflammation [43]. A peritoneal inflammation is established when the uterus fragments implanted in the peritoneum, leading to the degranulation of mast cells, production of ROS, PGs, chemokines and cytokines in the peritoneal fluid. Increased inflammatory actors in the endometrium, peritoneal fluid and serum of patients have been recently described in several studies and defined as lesion inflammation [48,49,50]. CBD administration reduced lesion inflammation as displayed by the increased Ikb-α and reduced COX2 cytosolic expressions and reduced NF-kB nuclear localization in the harvested lesions. Moreover, it strongly reduced peritoneal inflammation, as showed by the decreased TNF-α, PGE_2_ and IL-1β levels in the peritoneal fluid.

The immune response activated by the inflamed peritoneum may also contribute to directly activate nerve endings and induce positive feedback, further increasing proinflammatory mediator production. This neurogenic inflammation intensifies the painful stimuli transduced to the spinal cord, inducing chronic pelvic pain and central sensitization [51,52]. 

Mast cells play a key role in this contest. They could migrate from the nerve fibers, where they reside, to the spinal cord, to modulate neural activity and nociception [53]. Mast cell degranulation leads to the release of neuro-sensitizing and pro-inflammatory mediators [54,55,56]. CBD was able to down regulate mast cells migration and degranulation microglia in the spinal cord and in turn the expression levels of the pain-related pro-inflammatory mediators c-FOS and NGF. Mast cells infiltration also relates with the increased neuroinflammation and glial activation, which were assessed by Iba-1 and GFAP expressions. CBD administration significantly prevented both microgliosis and astrogliosis in spinal cord tissues. Well in line with these data, CBD administration from the behavioral point of view strongly reduced visceral sensitization altered pain threshold. 

The current research has some limitations. The employed endometriosis model was applied by transplanting normal rat uterine tissue into the abdominal cavity of another rat. It does not accurately represent the pathogenesis of human endometriosis [57]. However, rat models have a long history of being widely used in endometriosis research and have also been validated as a model that depicts the pathology dynamics [58]. In future experiments, it would be interesting to study the lesions for a longer period of time.

## 4. Materials and Methods

### 4.1. Animals

Sprague–Dawley rats (8–10 weeks old) (Envigo, Milan, Italy) were used in this research. The University of Messina Review Board for animal care (OPBA) approved the study (897/2021-PR). All animal experiments agree with the new Italian regulations (D.Lgs 2014/26), EU regulations (EU Directive 2010/63).

### 4.2. Endometriosis Induction

Animals were randomly assigned to two groups, donor or recipient. Donor rats were injected intraperitoneally with 10 IU PMSG to induce similar estrogen levels between various animals. The animals were euthanized 41 h later by progressive CO_2_ asphyxia. The uterus was removed through a midline incision and washed in PBS before extrauterine tissue, including ovary and oviduct, was removed under a dissecting microscope. A longitudinal incision was made from one horn to the other. Tissue was then transferred to a 1.5-mL centrifuge tube containing fresh PBS and minced with dissecting scissors [52]. Minced tissue from all donors was pooled, and the volume was adjusted to the equivalent of one uterus/500 µL of PBS. Recipient rats were injected intraperitoneally with the equivalent of tissue from one uterus in 500 µL of PBS (1:1 donor/recipient ratio) along the midventral line using an 18-gauge needle. The tissue was injected intraperitoneally to allow the development of lesion in the intraperitoneal region. The disease was allowed to establish for 7 days.

### 4.3. Experimental Groups

Recipient animals were randomly divided into the following groups (*n* = 30):

(1) Endometriosis group: animals were subjected to the experimental protocol as already described, and vehicle (ethanol/Tween 80/0.9% saline (3:1:16)) was orally administered on the 7th day and for the next 7 days;
(2) Endometriosis+CBD group: animals were subjected to the experimental protocol as already described, and CBD at the dose of 10 mg/Kg was orally administered on the 7th day and for the next 7 days;
(3) Sham group: animals were subjected to the same experimental protocol but they were intraperitoneally injected with the equivalent volume of phosphate buffered saline (PBS) along the midventral line instead of endometrial tissue.

CBD dosage was chosen based on previous experimental works [59,60]. 

Fourteen days from the endometriosis induction abdominal high-frequency ultrasound (hfUS) examination and behavioral analyses were performed. Animals were then euthanized by progressive CO_2_ asphyxia and laparotomy was performed to evaluate and collect the endometrial lesions. Additionally, an L4–L6 area of spinal cord tissues was harvested to evaluate the pain-related pro-inflammatory mediators expression. 

### 4.4. Abdominal High-Frequency Ultrasound

The hair in the ventral portion of the abdomen was carefully clipped from a point approximately 1 cm cranial to the xyphoid cartilage to the caudal-most part of the pubis. Alcohol and coupling gel were applied to the skin. To minimize interoperatorvariability, all ultrasonographic examinations were performed by the same operator (FM). Ultrasonographic examinations were performed using an Esaote MYLAB OMEGA VET (Esaote Italia, Milan, Italy) on sedated rats positioned in dorsal recumbency. Abdominal B-mode was performed with a High Frequency Linear array (4–15 MHz) transducer. The transverse and longitudinal scanning planes were used for evaluation of different abdominal structures. During the procedure, animals were anesthetized by 2% isoflurane. Measurements were performed offline (Esaote workstation) by a reader blinded to the condition of the rat [61]. 

### 4.5. Behavioral Analyses

#### 4.5.1. Open Field Test

A squared open field area was employed to evaluate exploratory behavior and locomotor activity [62,63]. After 1 min of conditioning, each rat was observed for 5 min starting from a corner of the area. After each test, the apparatus was cleaned with a solution of 20% ethanol. The recorded parameters were: spontaneous locomotion, identified as number of animal crossings with four legs, number of entries in central square and time spent in the central square (in seconds).

#### 4.5.2. Hot Plate

The answer to thermal stimuli was evaluated by the hot plate test. A hot surface (53.0 ± 0.1 °C) was employed and a cut of 45 s was established [64].

#### 4.5.3. Elevated plus Maze Test

The elevated plus maze apparatus employed [65] is composed of a central square connected to two closed arms and two open arms. Each rat was evaluated for 5 min. After each test, the apparatus was cleaned with a solution of 20% ethanol. For cleaning the apparatus after each analysis, a solution of 20% ethanol was used. The number of entries open arms and the time spent in it were reported as the %.

#### 4.5.4. Acetic-Acid-Induced Abdominal Contractions

The animals were tested for 20 min after an intraperitoneal injection of 0.6% acetic acid and the number of acid-induced writhes was recorded [52]. 

### 4.6. Reduced GSH Levels

Reduced GSH levels were evaluated in endometriosis lesions using a microplate reader at 412 nm as already described [66,67]. 

### 4.7. Lipid Peroxidation

The TBARS test was employed to evaluate the lipoperoxidation. The levels of MDA were assessed using a microplate reader at 535 nm as already described [68,69].

### 4.8. SOD Activity

SOD activity was determined as already shown and expressed as U/g protein [54,70,71].

### 4.9. Enzyme-Linked Immunosorbent Assay (ELISA)

IL-1β, TNF-α and PGE_2_ and levels were determined in peritoneal fluids using an ELISA kit (BioLegend, San Diego, CA, USA; R&D Systems, Milan, Italy) [72,73]. 

### 4.10. Histological Examination

For the histological analysis, endometriosis explants were fixed in buffered formaldehyde solution, dehydrated and embedded in Paraplast [74]. Tissue slides were stained with H&E and evaluated using a Leica DM6 microscope (Leica Microsystems SpA, Milan, Italy) [75]. Histopathologic scores were evaluated with the formula P (persistence of epithelial cells in the explants) × I (intensity of glands) as already described [76]. P: 3 = well-preserved epithelial layer, 2 = moderately preserved epithelium with leukocyte infiltrating, 1 = poorly preserved epithelium (occasional epithelial cells only), and 0 = no epithelium; I: from 0 (no glands) to 3 (abundant glands). Lesion volume was calculated according to the formula: V = (length × width^2^) × 0.5. [76]. Explants fibrosis was evaluated by the Masson trichrome staining (Bio-Optica, Milan, Italy) [77,78]. Mast cell analyses were performed by Toluidine blue staining employed to evaluate mast cells recruitment [79].

### 4.11. Immunohistochemical Analysis

Immunohistochemical localization of anti-GFAP (sc-33673) or anti-iba-1 (sc-32725) was performed in the spinal cord as already described [80,81]. All sections were incubated with the primary antibodies, then washed with PBS and treated as previously reported [82,83]. Stained sections were observed using a Leica DM6 microscope (Leica Microsystems SpA, Milan, Italy). The histogram profile is related to the positive pixel intensity value obtained [84].

### 4.12. Western Blot Analysis

Lesion samples and spinal cord tissues were homogenized and Western blots were performed as already described [85,86]. Specific primary antibody anti-Nox-1 (PA5-103220), anti-Nox-4 (PA5-72816), anti-MMP-9 (sc-13520), anti-iNOS (sc-7271), anti-TGF-β (sc-130348), anti-Ikb-α (sc-1643), anti-NF-kB (sc-8008), anti-COX2 (sc-376861), anti-c-FOS (sc-166940) and anti-NGF (sc-32300) was mixed in 5% *w*/*v* nonfat dried milk solution and was incubated overnight. Afterward, blots were incubated with peroxidase-conjugated bovine anti-mouse IgG secondary antibody or peroxidase conjugated goat ant-irabbit IgG (Jackson Immuno Research, Milan, Italy) for 1 h at room temperature [62]. Membranes were also blotted with the antibody against β-actin or lamin B1. Signals were detected with enhanced chemiluminescence detection system reagent (Super-SignalWest Pico Chemiluminescent Substrate) [87]. The relative expression of the protein bands was quantified by densitometry with Bio-Rad ChemiDoc XRS software (Bio-Rad, Milan, Italy) and standardized to β-actin or lamin B1 levels. Images of blot signals were imported to analysis software (v2003, Image Quant TL).

### 4.13. Statistical Evaluation

All values are expressed as mean ± standard error of the mean (SEM) of N observations. For in vivo studies, N represents the number of animals used. The results were analyzed by *t*-test when comparing two groups while we used the one-way ANOVA followed by a Bonferroni post hoc test for multiple comparisons. A *p*-value of less than 0.05 was considered significant. 

## 5. Conclusions

In conclusion, this paper focuses the attention on the CBD effect on endome-triosis, using an in vivo model. The data collected underlined its role in the upregulation of the ROS scavenging (SOD) enzymes and endogenous antioxidant systems (GSH) and downregulation of ROS producing (Nox) enzymes. These downregulations also link with anti-inflammatory and anti-proliferative and effects at the lesion size and in the peritoneal fluids. CBD administration also reduces the neurogenic inflammation by decreasing mast cells infiltration and degranulation in the spinal cord. Moreover, it reduced expression of the related neuro-sensitizing mediators, leading to a reduction of the astrocytes and mi-croglia activation. All these effects definitely are responsible of the reduced pain behavior and visceral sensitization. 

## Data Availability

The data presented in this study are available on request from the corresponding author.
